# Primary urachal leiomyosarcoma: a case report and literature review of clinical, pathological, and medical imaging features

**DOI:** 10.3389/fonc.2023.1228178

**Published:** 2023-08-17

**Authors:** Jing Yan, Hongwei Li, Gaowu Yan, Qing Duan, Chunyan Tang, Morgan A. McClure, Anup Bhetuwal, Yong Li, Ling Yang, Ruyi Li, Gangcheng Tan, Bo Feng

**Affiliations:** ^1^ Department of Radiology, Suining Central Hospital, Suining, China; ^2^ Department of Radiology, The Third Hospital of Mianyang, Sichuan Mental Health Center, Mianyang, China; ^3^ Department of Radiology and Imaging, Institute of Rehabilitation and Development of Brain Function, The Second Clinical Medical College of North Sichuan Medical College, Nanchong Central Hospital, Nanchong, China; ^4^ Sichuan Key Laboratory of Medical Imaging, Department of Radiology, Affiliated Hospital of North Sichuan Medical College, Nanchong, China; ^5^ Department of Pathology, Suining Central Hospital, Suining, China; ^6^ Department of Radiology, Lixian People’s Hospital, Aba, China

**Keywords:** urachus, urachal tumor, leiomyosarcoma, computed tomography, case report

## Abstract

**Background:**

Urachal tumors are exceedingly rare, and adenocarcinoma is the most common malignant urachal neoplasm. Here, an especially rare patient of primary urachal leiomyosarcoma from our hospital was reported, and only five patients have been reported thus far since 1981.

**Case description:**

A 24-year-old man was admitted due to urinary tract symptoms. Both urogenital ultrasonography and contrast-enhanced computed tomography showed a mass at the dome of the urinary bladder. Laparoscopic surgical resection was performed, and histopathologic examination of the mass confirmed the diagnosis of urachal leiomyosarcoma. No recurrence was noted after one and a half years.

**Conclusions:**

Because the leiomyosarcoma located in the extraperitoneal space of Retzius and may manifest with nonspecific abdominal or urinary symptoms, early and definitive preoperative diagnosis is challenging. Partial cystectomy with complete excision of the urachus is recommended. Because only a few patients have been recorded, clinical outcomes and recurrence risks are difficult to assess.

## Introduction

The urachus is a ductal remnant that arises embryologically, originating from the involution of the allantois and cloaca and extending between the bladder dome and the umbilicus (1). The tubular urachus normally involutes before birth remaining as a fibrous band with no known function (2). It is located in the loose connective tissue between the transversalis fascia and parietal peritoneum. Patients with urachal disease are usually asymptomatic because of its anatomical characteristics. Urachal diseases are usually found in conjunction with an infection or in urinary or other past abdominal and pelvic symptoms. The detection rate of urachal leiomyosarcomas is very low. Furthermore, urachal carcinoma accounts for < 1% of all bladder tumors, and approximately 80% of urachal carcinomas (UrC) are adenocarcinomas (3). We report a particularly rare patient of urachal leiomyosarcoma with a literature review conducted to shed more light on this disease (**Table 1**) (4–8).

**Table 1:** Clinical data of urachal leiomyosarcoma in the literature.

Reference	Year	Author	Number of cases	Sex	Age (years)	Clinical presentation	Imaging method	Location	Size (cm)	Imaging feature	Pathological type	Therapeutic method	Prognosis
(4)	1981	Noyes	1	Male	28	Dysuria, urgency, and frequency	Cystoscopy	The dome of the bladder	NA	NA	Low-grade leiomyosarcoma	A partial cystectomy with 2.0 cm margins and excision of the urachus	No evidence of recurrence at 3, 6, and 9 months.
(5)	1987	Jimenez	1	Male	15 months	Abdominal distension	Physical examination	Adhesion of the tumor to the anterior and lateral abdominal walls	NA	NA	Leiomyosarcoma	Resection	No clinical evidence of recurrence or metastasis at 10 months
(6)	2007	Kim	1	Male	12	Abdominal pain	Ultrasound examination	Superior to the bladder	5.9 × 3.7	A peripheral enhancing mass	Myxoid leiomyosarcoma	Partial cystectomy, partial omentectomy, and bladder irrigation	No evidence of recurrence at 6 months
(7)	2012	Saied	1	Female	21	Hemorrhagic shock	CT	At the dome of the urinary bladder inferiorly	19.5 × 8.9	A large heterogeneous complex mass	Low-grade leiomyosarcoma	Resection	No evidence of recurrence at 4 years
(8)	2022	Tong	1	Male	40	Dysuria, urinary frequency, and urgency	Urogenital US examination	Above the bladder	4.5 × 3.7	An oval mass with mild to moderate inhomogeneous enhancement	Low-grade leiomyosarcoma	Open surgical resection	No evidence of recurrence or metastasis at 53 months
Our patient	2022	Yan	1	Male	24	Urinary frequency, pollakiuria, and dysuria	US	At the dome of the bladder	2.7 × 2.1 × 2.1	Heterogeneous enhancement of the mass and thickened bladder wall	Leiomyosarcoma	A partial cystectomy and excision of the urachus	No recurrence at 3 months

CT, Computed tomography; US, Ultrasonography; NA, Not available.

## Case presentation

**Figure 1:**
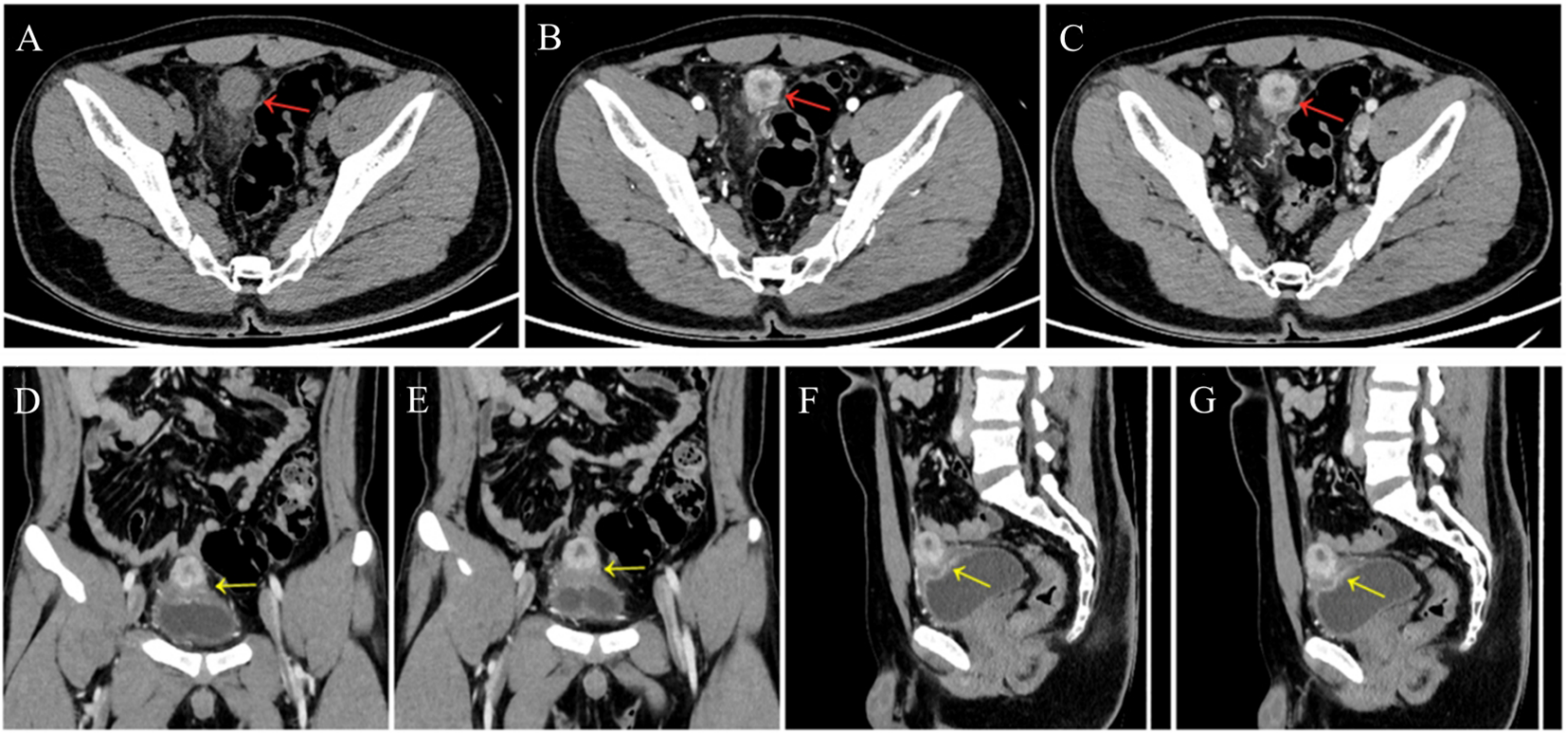
A 24-year-old man presented a slightly hypodense mass (red arrows) at the dome of the bladder **(A)**, at arterial phase **(B, D, F)** and venous phase **(C, E, G)**; contrast-enhanced CT scan showed peripheral enhancement of the mass and no enhancement of necrosis. Coronal **(D, E)** and sagittal **(F, G)** images showed the thickened bladder wall (yellow arrows).

**Figure 2:**
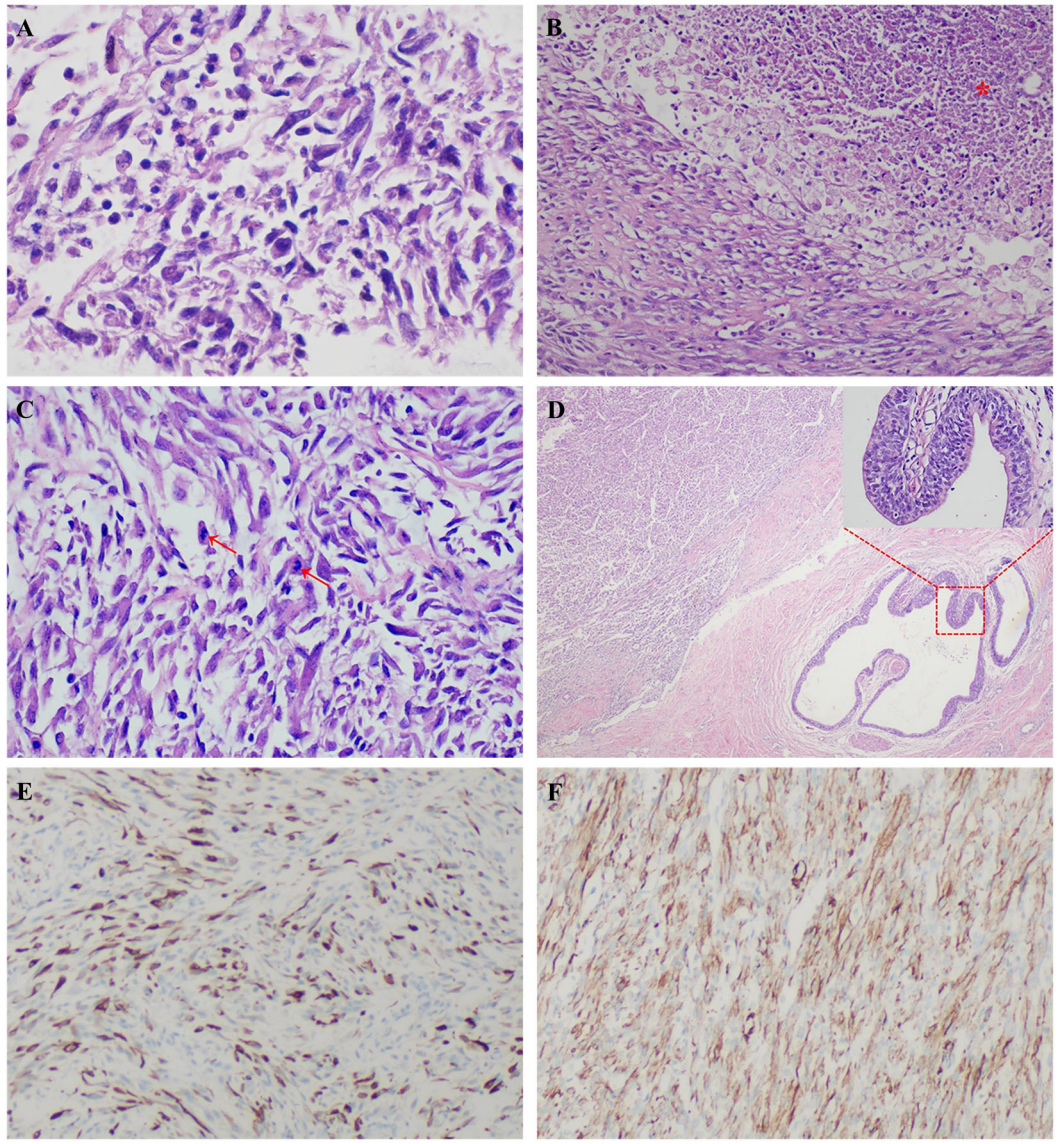
A 24-year-old man was diagnosed with a primary urachal leiomyosarcoma. Microscopic view showed irregular intersecting bundles of spindle cells with mild cell pleomorphism **(A)**, HE × 600), which indicating a well differentiated leiomyosarcoma. There was necrosis observed in this tumor **(B)**, red star, HE × 200). Some of the nuclei showed atypia **(C)**, red arrows, HE × 400). Some urachal remnants were identified within the tumor tissue that near the abdominal wall side **(D)**, HE × 40), covered with columnar and transitional epithelium cells **(D)**, red square, HE × 400). Immunohistochemical stains showed positivity for desmin **(E)**, × 100) and smooth muscle actin (SMA, **(F)**, ×100), compatible with leiomyosarcoma.

**Figure 3:**
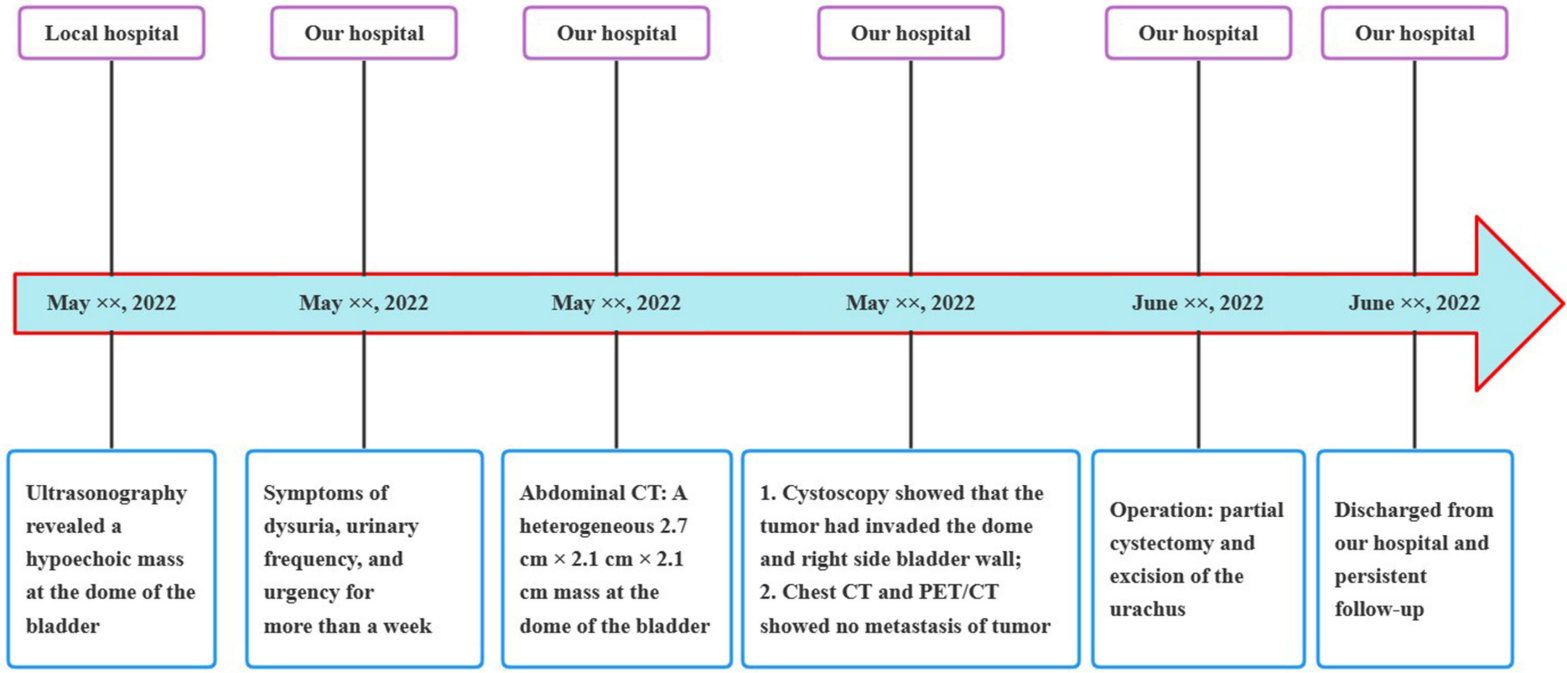
Medical timeline of the patient with primary urachal leiomyosarcoma. CT, computed tomography; PET/CT, positron emission tomography/computed tomography.

**Figure 4:**
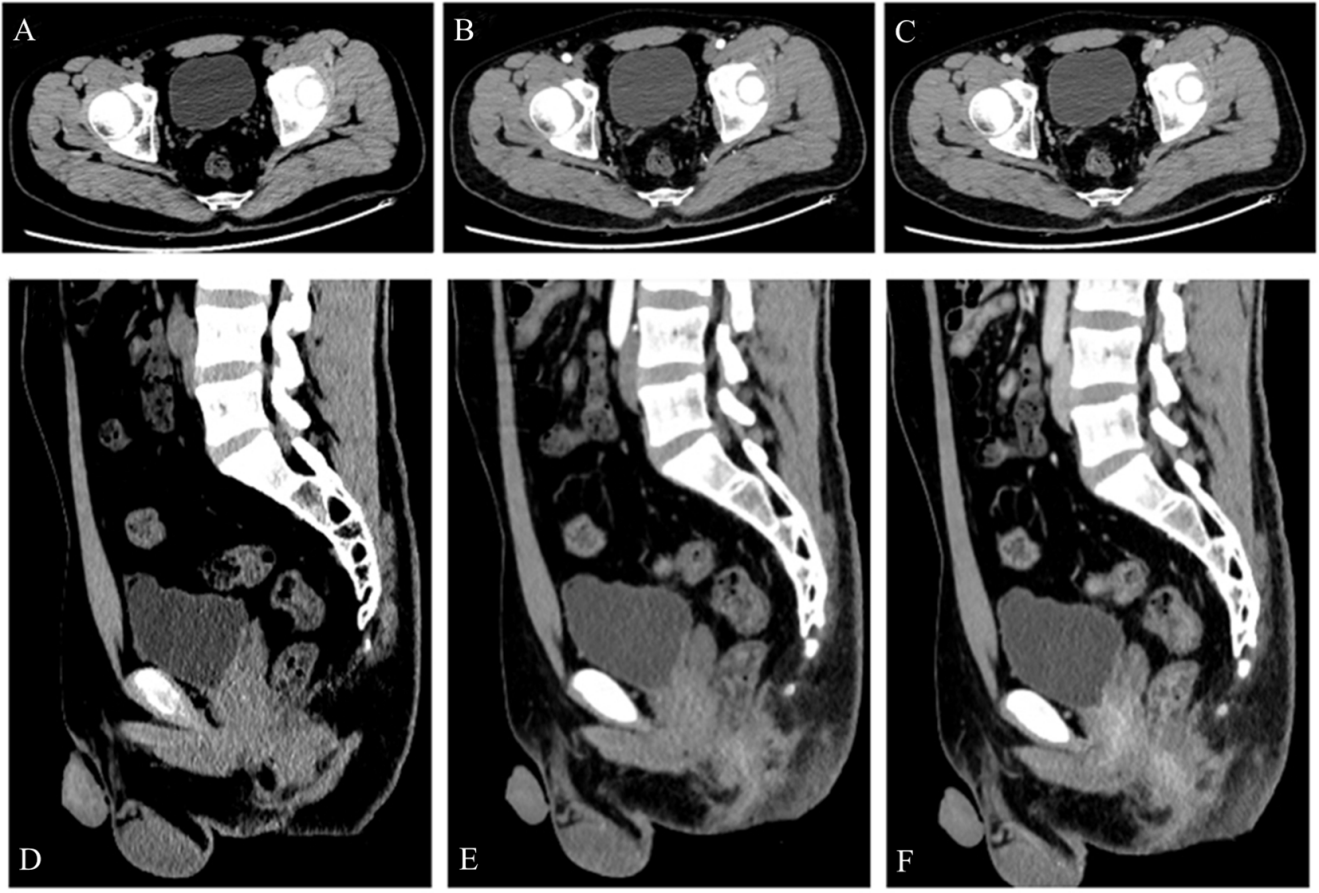
A 24-year-old man was diagnosed with a urachal leiomyosarcoma. One and a half years after surgery, contrast-enhanced CT examination **(A–F)** showed no recurrence of the tumor.

A 24-year-old man manifested the symptoms of dysuria, urinary frequency, and urgency for more than a week. Urogenital ultrasonography (US) revealed a hypoechoic mass at the dome of the bladder. The patient was treated with anti-inflammatory therapy and discharged from the local hospital after improvement. Thereafter, the patient sought consultation at our hospital for further evaluation. Contrast-enhanced abdominal computed tomography (CT) showed a heterogeneous 2.7 cm × 2.1 cm × 2.1 cm mass at the dome of the bladder (**Figure 1**). The boundary of the mass was obscure, and the adjacent bladder wall was irregularly thickened. Inhomogeneous enhancement of the mass revealed a thickened bladder wall in the arterial and venous phases. Putative clinical diagnosis included urachal adenocarcinoma, squamous cell carcinoma, urachal cystic mass, or abscess. Cystoscopy showed that the tumor had invaded the dome and right-side bladder wall, and the biopsy revealed some normal urothelium. Chest CT and whole-body positron emission tomography/computed tomography (PET/CT) showed no metastasis of tumor. After sufficient preoperative preparation, a partial cystectomy and excision of the urachus were scheduled for both definitive diagnosis and therapeutic purposes. The intraoperative findings are: the tumor had infiltrated the whole urachus and adhered to the peritoneum; the tumor was located at the dome of the bladder, infiltrating the bladder wall, with a size of 4.2 cm × 3.5 cm × 3.5 cm; the tumor was fish-meat like appearance; no necrosis or bleeding was noted. Pathological and immunohistochemical examinations (desmin (+), SMA (+), Bcl-2 (–), MyoD1 (-), myogenin (-), CD117 (-), CD34 (-), DOG1 (-), S100 (-), PHH3 (+, with mitotic activity), Ki-67 (+, 20%)) of the mass confirmed the diagnosis of a leiomyosarcoma originating from the urachus (**Figure 2**). Our diagnostic criteria for interpreting a carcinoma as of urachal origin are (9, 10): (a) tumor located in the dome or anterior bladder wall; (b) tumor growth in the bladder wall; (c) absence of atypical intestinal metaplasia and cystitis/glandularis beyond the dome/anterior wall; (d) absence of a urothelial bladder neoplasia; (e) exclusion of a primary adenocarcinoma of a different origin. Some urachal remnants were identified within the tumor tissue that near the abdominal wall side, covered with columnar and transitional epithelium cells. The result is in accordance with the consensus statement by the Canadian Urological Association and Genitourinary Medical Oncologists of Canada (10). In the consensus, urachal remnants were reported in only 15%-62.5% of patients; their absence should not be considered exclusionary for a urachal primary. The patient was discharged after a week (**Figure 3**), and no recurrence was noted at one and a half years (**Figure 4**).

## Discussion

### Clinical features

The urachus is a 3.0 - 10.0 cm tubular structure extending from the allantois to the early fetal bladder. During embryonic development, the upper part of the allantois will be reduced gradually. After birth, it is replaced by fibromuscular tissue, also known as the median umbilical ligament. In patient of partial obliteration of the urachus, various diseases including fistula, diverticulum, or tumor may occur. The tumor primarily occurs in males and is usually localized in the bladder dome or anterior wall. Urachal adenocarcinoma is the most common pathological type (up to 80%), with an occasional occurrence of squamous cell carcinoma (1). The patient of urachal leiomyosarcoma is extremely rare. Urachal leiomyosarcomas are typically silent because of their extraperitoneal location, but initial symptoms of infection, local invasion, or metastatic disease may include hematuria, irritative voiding symptoms, and suprapubic pain (2, 11). Prognosis is generally poor due to the anatomic location, late onset of symptoms, and high risk of metastasis. Tumor grade and surgical margin are the most important prognostic factors.

### Histologic and pathologic features

Leiomyosarcoma is a malignant neoplasm characterized by smooth muscle differentiation. The World Health Organization (WHO) proposed a histological classification of leiomyosarcoma which is divided into myxoid and epithelioid leiomyosarcoma (12). Leiomyosarcomas of the retroperitoneal region, digestive tract, and uterus are more commonly observed. Urachal leiomyosarcoma derived from the smooth muscle tissue of the urachus is considered extremely rare. Similar to leiomyosarcoma in other parts of the body, urachal leiomyosarcomas usually demonstrate noticeable interlacing bundles of muscle fascicles with spindle cells that have cigar-shaped nuclei and abundant eosinophilic cytoplasm. Most tumors have pleomorphism, necrosis, and a high frequency of mitoses. Immunohistochemical studies showed that tumor cells are immunopositive to smooth muscle actin and desmin, but are immunonegative to keratin, S-100, HMB-45, MyoD1 and myogenin. Myogenic markers such as actin and desmin are also expressed in rhabdomyosarcoma and non-myogenic sarcoma. MyoD1 and myogenin are useful in deafferenting leiomyosarcoma from rhabdomyosarcoma, as they are regulatory transcriptional factors expressed in the early stages of skeletal muscle differentiation. Most studies have confirmed that they are sensitive and specific immunohistochemical markers for rhabdomyosarcoma, and an overexpression of myogenin particularly facilitates the diagnosis (13). Therefore, when myogenin is negatively expressed, it is a very useful marker to help identify rhabdomyosarcoma. Expressions of MyoD1 and myogenin in the tumor of our patient both are negative. Leiomyosarcoma is usually graded according to the three-level classification standard of the French Federation of Cancer Centers Sarcoma Group. The local recurrence rate or metastasis is related to the tumor grade. Primary urachal leiomyosarcoma has no specific imaging findings. Therefore, the diagnosis of urachal leiomyosarcoma is often difficult and delayed. Moreover, the final diagnosis is confirmed through pathologic examination.

### Imaging diagnostic modalities

US is the preferred method in the detection of urachal leiomyosarcoma. US can show the urachal tract between the bladder dome and the umbilicus allowing localization of the tumor. The CT appearance of a leiomyosarcoma is not specific; necrotic or cystic change is usually observed and reveals no enhancement; therefore, it shows an uneven and avid enhancement on contrast-enhanced CT. On MRI, the urachal leiomyosarcoma is reported to be isointense on T1-weighted imaging (T1WI) and heterogeneously hyperintense on T2-weighted imaging (T2WI). A high signal intensity on T2WI is associated with fluid, necrosis, or mucin. The contrast-enhanced MRI findings are similar to that of the contrast-enhanced CT.

Recent studies demonstrate that ^18^F-fluorodeoxyglucose (FDG) PET/CT may be helpful for diagnosing primary and recurrent urachal adenocarcinoma (14). On ^18^F-FDG PET/CT, the primary tumor and metastasis are classified as ^18^F-FDG-avid (above the local background). In identifying the primary focus, FDG PET/CT does not seem to yield additional information compared with CT or MR. However, it may detect metastatic lesions not seen on other imaging modalities, causing changes in tumor stage and patient management (15).

### Staging

Currently, there are some different staging approaches (e.g., Sheldon, Mayo, and modified TNM staging systems) for urachal cancer, but none of them have been fully validated. The Sheldon staging system is complex and has eight categories, while the Mayo system is simpler and more proportional for patient distribution. Based on the Mayo system, urachal cancer is classified into four stages: Stage I, tumor confined to urachus and/or bladder; Stage II, tumor extending beyond the muscular layer of urachus and/or bladder; Stage III, tumor infiltrating the regional lymph node; and Stage IV, tumor infiltrating non-regional lymph nodes or distant sites (16). Therefore, our patient can be classified as the stage II of the Mayo system.

Compared with other modalities for staging of urachal cancer, MRI has more advantages in soft tissue resolution and multi-plane imaging capabilities. For primary UrC, MRI can provide the tumors’ location, size, morphology, extent of peritoneal tissue, and/or local structures beyond bladder invasion. For recurrent UrC, it can accurately evaluate recurrence and metastatic patterns. Studies have shown Mayo stage at MRI is highly consistent with pathology (17).

Notably, UrC are often mucinous in histology, and this can lead to low or absent ^18^F-FDG uptake due to hypocellularity of tumors which may limit the sensitivity of ^18^F-FDG PET/CT (18). Physicians and PET readers should realize the potential pitfall in trying to stage urachal cancer either in the context of primary or recurrent urachal tumors and consider combining with other imaging modalities such as MRI, to further evaluate mucinous malignancy (19).

### Differential diagnosis

Urachal carcinoma commonly occurs in the middle-aged and elderly people and primarily in men (20). Despite the fact that the urachus is lined by urothelium, more than 80% of UrC are adenocarcinomas (1). On US, Urachal adenocarcinomas usually appear as a midline soft-tissue mass over the apex of the bladder with complex echogenicity. On CT, the mass can be cystic, solid, or mixed. It usually appears as a midline mass near the bladder dome with areas of low attenuation representing mucin content. Approximately 50% - 70% of tumors showed calcification (21). On MRI, sagittal images may be the best modality for the evaluation of urachal neoplasms. The solid part of the lesion appeared as isointense on T1WI and T2WI compared to soft tissue. There is also a high signal intensity area on T2WI images that is associated with mucin content. In gadolinium diethylenetriamine pentaacetic acid (Gd-DTPA) enhancement scan, the solid part is enhanced, and the area of T2WI hyperintensity will demonstrate enhancement of glandular tissue which can be differentiated from fluid or necrosis (1). In recent years, MRI has become one of the most used modalities in bladder cancer evaluation (22). Most of the literature, though, on the MR imaging of UrC are case reports and reviews. The research on clinical applications of MRI is limited. A recent study (17) showed that for primary UrC, MRI is able to accurately identify the tumor location (in relation to the bladder), morphology, maximum diameters, invasion of adjacent structures, Mayo stage on MRI, and multiparametric MRI features of urachal tumor; for recurrent UrC, MRI can effectively evaluate the local tumor recurrence (location and MRI features), detect lymph nodes, and distant metastasis (23). Therefore, MRI can offer effective assessment to preoperative staging, detection of local recurrence, and aid in the evaluation of the tumor response in the treatment (24).

A detailed description of some urachal neoplasms is summarized in **Table 2**.

**Table 2:** Clinical and imaging features of urachal leiomyosarcoma, common benign or malignant urachal neoplasms.

Urachal neoplasms	Benign or malignant	Percentage (%)	Susceptible age	Presenting symptoms	Morphological variation	US	CT	MRI		Enhancement
								T1WI	T2WI	
Villous adenomas	Benign	NA	50 years old or older	Urinary mucus	Unilocular cystic	Hypoechoic	Cystic mass	Hypointense	Predominantly hyperintense	Non-enhancing
Mucinous cystadenomas	Benign	NA	Older than 50 years with a male predominance	Hematuria, irritative voiding symptoms, and mucusuria	Multilocular cystic	Inhomogeneous hypoechoic mass	Heterogeneous cystic lesion	Hypointense	Hyperintense	Cystic walls and internal septa with slight enhancement
Leiomyoma	Benign	NA	Older than 45 years	Palpable mass, urinary frequency	Irregularly shaped solid tumor	Anechoic or hypoechoic mass	Heterogeneous, predominantly hypodense lesion	NA	NA	Mild enhancement
Hamartoma	Benign	NA	Adults	Irritative bladder symptoms	Solid, cystic mass	Hypoechoic	Isodense or mixed density	Isointense or slightly hyperintense	Heterogeneously hyperintense	Significantly enhancing
Leiomyosarcoma	Malignant	NA	All ages	Hematuria, dysuria, urinary frequency, and urgency	Fleshy, with cystic spaces, necrosis	Hypoechoic	Not specific; necrotic or cystic change is usually seen	Isointense	Heterogeneous hyperintensity	Uneven and avid enhancement
Inflammatory myofibroblastic tumors	Uncertain malignant potential	NA	Children and young adults	Suprapubic pain	Solid cystic mixed mass	Heterogeneous mass	Mixed and crystal low density	Low-to-moderate signal intensity	Slight high signal intensity	Peripherally enhancing, blurred edges
Adenocarcinomas	Malignant	80	Middle-aged and elderly men	Hematuria, mucusuria	Ulcerated or polypoid mass	Mixed echogenic mass	Cystic, solid, or mixed, 50%-70% with calcification	Isointense	Isointense, hyperintense	centrally non-enhancement and peripheral enhancement
Urothelial	Malignant	10	Older than 65 years	Hematuria	Papillary mass	Focal papillary projection, hypoechoic	Soft tissue nodule	Isointense	Isointense	Early enhancement
Squamous	Malignant	3	Middle-aged and elderly men	Hematuria	Soft tissue mass	Hypoechoic	Papillary soft tissue mass or plaque-like thickening	Isointense	Isointense	Mild enhancement

US, Ultrasonography; CT, Computed tomography; MRI, Magnetic resonance imaging; T1WI, T1-weighted imaging; T2WI, T2-weighted imaging; NA, Not available.

### Treatment and prognosis

The recommended treatment of urachal carcinoma is partial cystectomy and excision of the urachus including a peritoneal cuff which is the first treatment intervention for most patients of urachal leiomyosarcoma that have been reported (4). As a result of the lack of data on urachal leiomyosarcomas, adjuvant chemotherapy and radiotherapy for urachal leiomyosarcoma are controversial and the clinical outcomes and prognosis are difficult to assess (25). A previous study reported a spontaneously ruptured urachal myxoid leiomyosarcoma in an adolescent patient who was treated with radiation therapy and alternating adjuvant chemotherapy (vincristine, doxorubicin and cyclophosphamide regimen, vincristine, dactinomycin and cyclophosphamide regimen) (6). However, no long-term follow-up data were reported in that study. In addition, neoadjuvant chemotherapy for bladder leiomyosarcoma may provide reference for neoadjuvant regimens are doxorubicin, ifosfamide, cisplatin, adriamycin, and vincristine (26).

The patient in our report was not treated with adjuvant chemotherapy or radiotherapy, and no recurrence was noted at one and a half years. We think that urachal tumors are not particularly sensitive to radiotherapy. As a result, radiotherapy can be considered for patients unfit for surgery, postoperative positive margins, and incurable disease (palliative radiation). For chemotherapy, we suggest that it should not be recommended on a routine basis, as types of treatment used are heterogeneous and the benefits are unclear. Postoperative chemotherapy may be considered due to positive margins, lymph node or peritoneal involvement, or unresected umbilicus and a high likelihood of relapse (10). Finally, cystoscopic evaluation has the tendency to cause local recurrence. Thus, imaging examination can be used to evaluate whether there are new or recurrent lesions after surgery.

### Follow-up

As a result of the lack of data on urachal leiomyosarcomas, follow-up scheme of this entity is not clear in the literature (10). For the patient in our study, we referred to the follow-up scheme for urachal carcinoma and bladder cancer (9, 27–30). Specifically, we recommended that the patient a regular follow-up after the operation including visits to an outpatient clinic every 3 months for the first 2 years and every 6 months for the following years. We also advised the patient to undergo routine blood and biochemical analyses, physical examinations, chest radiographies, and US/CT/MRI examinations of the abdomen and pelvis, as appropriate. When there was any evidence of suspected recurrence, chest, abdominal, and pelvic CT scans; brain MRI; bone scintigraphy; and PET/CT might be performed, as appropriate.

## Conclusions

Leiomyosarcoma is a type of soft-tissue sarcoma usually occurring in the soft tissues of the extremities and trunk. It is aggressive and spreads hematogenously which are characteristics of a malignant neoplasm. Primary leiomyosarcoma of the urachus is extremely rare, and it tends to be easily misdiagnosed. Understanding the embryological basis of these urachal disorders and their imaging features coupled with histopathological, examination is crucial for the correct diagnosis and management.

## Patient perspective

Our patient reported the following about his disease: “Throughout my clinical care, I have been concerned that my disease would come back. I now have an abdominal ultrasonography every three months to keep track of my disease. Fortunately, I am now in good health. Thanks are given to the doctors and nurses who have taken care of me and helped me.”

## Data availability statement

The original contributions presented in the study are included in the article/supplementary material. Further inquiries can be directed to the corresponding author.

## Ethics statement

The studies involving human participants were reviewed and approved by Institutional Ethics Committee of the Suining Central Hospital. The patients/participants provided their written informed consent to participate in this study.

## Author contributions

JY, QD, and GY participated in the study design and writing of the manuscript. JY, QD, GY, CT, HL, and YL participated in clinical data collection and analysis, and carried out the interpretation of the CT images. LY performed the pathological and immunohistochemical examinations. GY, MM, AB, RL, GT and BF all revised the paper critically for intellectual content. All authors contributed to the article and approved the submitted version.
